# CXCR6^+^CD4^+^ T cells promote mortality during *Trypanosoma brucei* infection

**DOI:** 10.1371/journal.ppat.1009968

**Published:** 2021-10-06

**Authors:** Gongguan Liu, Osama Abas, Ashley B. Strickland, Yanli Chen, Meiqing Shi

**Affiliations:** Division of Immunology, Virginia-Maryland College of Veterinary Medicine and Maryland Pathogen Research Institute, University of Maryland, College Park, Maryland, United States of America; INRS - Institut Armand Frappier, CANADA

## Abstract

Liver macrophages internalize circulating bloodborne parasites. It remains poorly understood how this process affects the fate of the macrophages and T cell responses in the liver. Here, we report that infection by *Trypanosoma brucei* induced depletion of macrophages in the liver, leading to the repopulation of CXCL16-secreting intrahepatic macrophages, associated with substantial accumulation of CXCR6^+^CD4^+^ T cells in the liver. Interestingly, disruption of CXCR6 signaling did not affect control of the parasitemia, but significantly enhanced the survival of infected mice, associated with reduced inflammation and liver injury. Infected CXCR6 deficient mice displayed a reduced accumulation of CD4^+^ T cells in the liver; adoptive transfer experiments suggested that the reduction of CD4^+^ T cells in the liver was attributed to a cell intrinsic property of CXCR6 deficient CD4^+^ T cells. Importantly, infected CXCR6 deficient mice receiving wild-type CD4^+^ T cells survived significantly shorter than those receiving CXCR6 deficient CD4^+^ T cells, demonstrating that CXCR6^+^CD4^+^ T cells promote the mortality. We conclude that infection of *T*. *brucei* leads to depletion and repopulation of liver macrophages, associated with a substantial influx of CXCR6^+^CD4^+^ T cells that mediates mortality.

## Author summary

We have previously shown that liver macrophages engulf African trypanosomes through complement receptor CRIg. However, the fate of liver macrophages following phagocytosis and their influence on subsequent T cell responses remain incompletely understood. In this study, we show that liver macrophages were depleted during infection of trypanosome parasites *T*. *brucei* and repopulated. The replenishment of liver macrophages was associated with a substantial recruitment of CXCR6^+^CD4^+^ T cells in the liver. Interestingly, infected CXCR6 deficient mice displayed an impaired recruitment of CD4^+^ T cells due to a cell-intrinsic defect of CXCR6 deficient CD4^+^ T cells. Importantly, CXCR6^+^CD4^+^ T cells promoted liver injury, resulting in the early mortality of infected mice. Collectively, those results demonstrate that liver macrophages undergo cell death and repopulation, accompanied by a substantial accumulation of CXCR6^+^CD4^+^ T cells that promote mortality during *T*. *brucei* infection.

## Introduction

The liver is the biggest internal organ with important functions of both metabolism and immunity [1]. Of note, the liver plays a prominent role in filtering of microbes circulating in the bloodstream through Kupffer cells (KCs) [2–6]. KCs are liver-resident macrophages that adhere to the endothelial cells of the liver sinusoids [1]. Like tissue resident macrophages in other organs, KCs are embryonic origin and self-renewing under steady condition [7–9]. Depletion of KCs by diphtheria toxin leads to a rapid repopulation of KCs by bone marrow monocytes [10,11]. In the infection setting, KCs infected by bacterial pathogen *Listeria monocytogenes* undergo rapid necroptotic death, followed by replenishment of the depleted KCs with circulating monocytes and subsequent tissue repair [12]. It remains poorly understood how this process affect adaptive immunes responses, particularly T cell responses in the liver following phagocytosis of bloodborne parasites by KCs.

African trypanosomes are bloodborne parasites that infect humans and animals. The parasites survive in the bloodstream, developing sophisticated mechanisms to escape host immune responses and causing death of the hosts if left untreated [13–15]. The liver is the major place to filter the parasites out of the bloodstream [16,17]. KCs plays an essential role in the clearance of the parasites through interaction of CRIg with complement components C3b/iC3b deposited on the parasite [4]. IFN-γ, secreted by multiple subsets of lymphocytes [18–20], has been shown to mediate protection [18,20–23]. However, excessive production of IFN-γ, particularly secreted by CD4^+^ T cells, mediates lethal liver pathology in mice during African trypanosome infections [24–26]. In this context, we have recently shown that IL-27 prevents liver pathology and is crucial for survival during African trypanosomiasis via downregulation of intrahepatic CD4^+^ T cell activation and its secretion of IFN-γ [27,28]. However, the molecular mechanisms regulating the accumulation of CD4^+^ T cells in the liver of mice infected with African trypanosomes remain elusive.

Interactions between chemokines and chemokine receptors are known to play a role in the recruitment and retention of leukocytes in infected and inflamed tissues. The chemokine receptor CXCR6, also referred to as STRL33, BONZO, or TYMSTR, was initially identified as a co-receptor for simian immunodeficiency virus and human immunodeficiency virus [29,30]. It is expressed on various subsets of lymphocytes including CD4^+^ T cells, CD8^+^ T cells, NKT cells, B cells, NK cells [31–33], and innate lymphoid cells [34]. The chemokine CXCL16 was originally described as a scavenger receptor of macrophages for phosphatidylserine and oxidized low-density lipoprotein [35] and is the only ligand of CXCR6 [32,36,37]. CXCL16 is expressed on the surface of a wide range of cells including macrophages [31]. The membrane-bound CXCL16 can be cleaved by the proteases, ADAM-10 or ADAM-17 [38], leading to the release of soluble form of CXCL16 which acts as a chemoattractant for CXCR6^+^ cells [32,39]. In this regard, a significant defect in the number of NKT cells and CD8^+^ T cells has been observed in the inflamed liver in the absence of CXCR6/CXCL16 signaling [39–43]. However, the role of CXCR6-CXCL16 axis during infection has not been well documented.

In this investigation, we found that infection by *Trypanosoma brucei* induced cell death of macrophages in the liver, leading to the repopulation of CXCL16-secreting macrophages, associated with accumulation of CXCR6^+^CD4^+^ T cells in the liver. Importantly, infected CXCR6 deficient mice displayed significantly enhanced survival compared to wild-type (WT) mice, characterized by reduced intrahepatic number of CD4^+^ T cells and diminished liver pathology. The reduction of CD4^+^ T cells in the liver was attributed to an intrinsic property of CXCR6 deficient CD4^+^ T cells. Adoptive transfer experiments demonstrated that CXCR6^+^CD4^+^ T cells promoted the mortality of infected mice. We conclude that CXCR6^+^CD4^+^ T cells mediate the early mortality during *T*. *brucei* infection.

## Results

### *T*. *brucei* infection induces depletion and repopulation of intrahepatic macrophages

**Fig 1:**
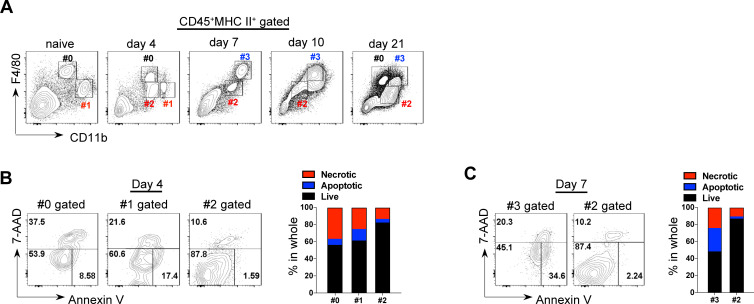
Depletion and repopulation of intrahepatic macrophages during *T*. *brucei* infection. **A** Flow cytometric analysis showing infection-induced dynamic shifts in the composition of intrahepatic macrophage subsets in wild-type (WT) mice. The subsets marked in the quadrant were identified based on the relative expression of F4/80 and CD11b within the CD45^+^MHCII^+^ gate. **B, C** The apoptotic and necrotic death of the indicated intrahepatic macrophage subsets in WT mice on day 4 (**B**) or 7 (**C**) post infection. (Left) Representative flow cytometric plots. (Right) Quantification of the percentages. n = 4 (**A-C**).

As liver is the major place for the clearance of trypanosome by macrophages, we first examined the dynamic composition of liver macrophages upon *T*. *brucei* infection. Given the heterogenicity in phenotype and origin, macrophage subsets here were defined based on their constitutive expression of MHC II and the relative intensity of F4/80 and CD11b [44]. As shown in Fig 1A, infection induced the loss and regain of F4/80^hi^CD11b^int^ macrophages, and the infiltration of F4/80^hi^CD11b^hi^ and F4/80^int^CD11b^int^ macrophages. To determine the nature of their dynamic fluctuation in population size, we measured cell death of macrophage subsets at different stages of infection. Annexin V and 7-AAD staining showed that the resident and the infiltrating F4/80^hi^ subsets were highly apoptotic or necrotic on day 4 (onset of the parasitemia) or day 7 (peak of the parasitemia) post infection (Fig 1B and 1C). By contrast, the infiltrating F4/80^int^CD11b^int^ macrophages were highly resistant to cell death (Fig 1B and 1C).

**Fig 2:**
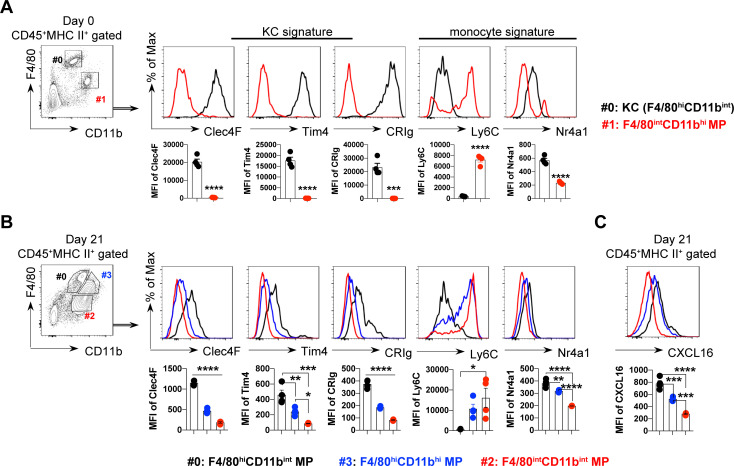
Characterization of liver macrophages during *T*. *brucei* infection. **A-C** The expression of phenotypic markers by the indicated intrahepatic macrophage subsets in WT mice on day 0 (**A**) or 21 (**B, C**) post infection. (Top) Representative flow cytometric analysis of gated macrophage populations (#0-3). (Bottom) Quantification of the expression using median fluorescent intensity (MFI) of gated macrophage populations (#0-3). n = 4, data are expressed as mean ± SEM from 2 independent experiments, compared by unpaired two-tailed *t* test (**A**) or one-way ANOVA with Tukey’s multiple comparisons test (**B, C**). Dots represent biological replicates. *p< 0.05, **p< 0.01, ***p<0.001, and ****p< 0.0001.

To characterize the liver macrophages, we defined the phenotype of the infiltrating macrophage subsets by referring to the signature markers of KCs and circulating monocytes. At steady condition, as expected, F4/80^hi^CD11b^int^ macrophages were the embryonic KCs (em-KCs) with an exclusive and intense expression of Clec4F, Tim4, and CRIg, but low expression of Ly6C (Fig 2A). During infection, F4/80^hi^CD11b^int^ macrophages resembled em-KCs’ phenotype with higher levels of Clec4F, Tim4, CRIg, and Nr4a1 but lower levels of Ly6C compared to F4/80^hi^CD11b^hi^ and F4/80^int^CD11b^int^ subsets (Fig 2B). Interestingly, repopulated F4/80^hi^CD11b^int^ macrophages displayed higher intensity of CXCL16 than F4/80^hi^CD11b^hi^ and F4/80^int^CD11b^int^ subsets (Fig 2C). We found that infection did not alter the expression of sheddase ADAME10 by F4/80^hi^CD11b^int^ macrophages (S1A Fig). However, compared to naïve mice, F4/80^hi^CD11b^int^ macrophages in infected mice exhibited enhanced expression of CXCL16 (S1B Fig).

Collectively, these results suggest *T*. *brucei* infection induced cell death and replenishment of liver macrophages.

### Substantial recruitment of CXCR6^+^CD4^+^ T cells in the liver

**Fig 3:**
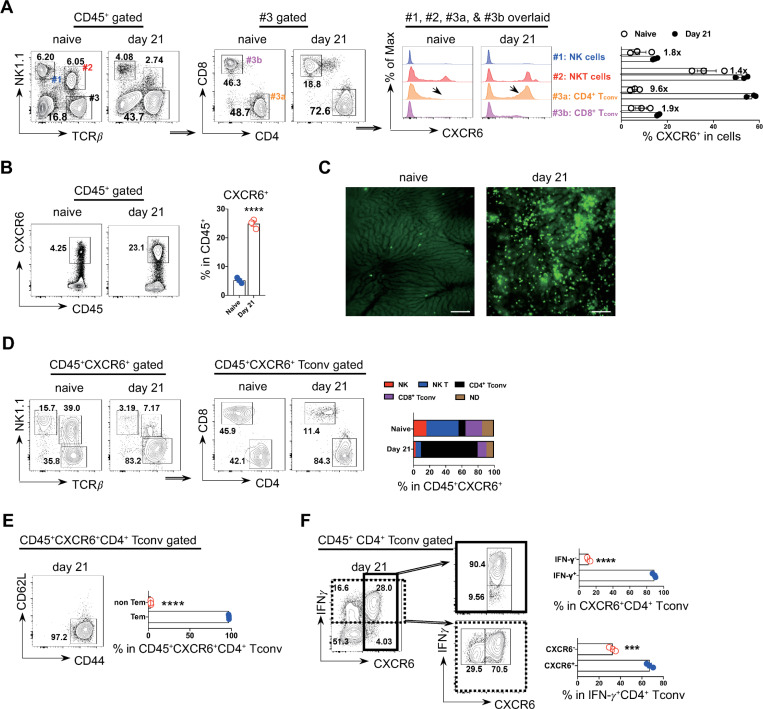
Accumulation of CXCR6^+^CD4^+^ T cells in the liver during *T*. *brucei* infection. **A** Flow cytometric analysis showing the sharpest increase of CXCR6^+^ events in intrahepatic CD4^+^ conventional T cells (Tconv) in WT mice upon infection. Numbers annotated within the bar graph indicate the folds of enhancement of the CXCR6^+^ events within distinct cell subsets upon infection. **B** The frequency of the CXCR6^+^ cells among total intrahepatic CD45^+^ leukocytes on indicated time-points of infection. (Left) Representative plots. (Right) Quantification of the frequency within the CD45^+^ cells. **C** Representative intravital images comparing the abundance of CXCR6-GFP cells in CXCR6^+/gfp^ mice between day 0 and day 21 post infection. CXCR6^+^ cells: green. Scale bar, 200 μm. **D** The frequency of the indicated cell subsets among total intrahepatic CXCR6^+^ cells on day 0 and day 21 post infection. (Left and middle) Representative plots. (Right) Quantification of the frequency within the CD45^+^CXCR6^+^cells. ND, not determined. **E** The frequency of CD44^+^CD62L^-^ cells (effector memory T cells, Tem) among intrahepatic CXCR6^+^CD4^+^ Tconv cells. (Left) Representative flow cytometric analysis. (Right) Quantification of the frequency. **F** Flow cytometric analysis and bar graph showing CXCR6^+^CD4^+^ T cells are exclusively IFN-γ producing cells and represent a major IFN-γ producing CD4^+^ T cell subset. n = 3, data are expressed as mean ± SEM from 2 independent experiments, compared by unpaired two-tailed *t* test (**B, E, F**). Dots represent biological replicates. ***p<0.001, and ****p< 0.0001.

Having shown the depletion and repopulation of liver macrophages, next we examined T cell recruitment to the liver during infection. Compared to naive mice, infected mice displayed higher frequency of T cells, particularly CD4^+^ T cells in the liver (Fig 3A). Moreover, the frequency of CXCR6^+^CD4^+^ T cells within total CD4^+^ T cells increased 9.6 folds following infection, the highest increase among NK cells, NKT cells, CD4^+^ and CD8^+^ T cells (Fig 3A). Next, we examined the composition of CXCR6^+^ cells in the liver. The frequency of CXCR6^+^ cells was significantly enhanced in infected mice as compared to naïve mice (Fig 3B). In vivo imaging showed more CXCR6-GFP cells accumulated in the liver of CXCR6^+/gfp^ mice following infection (Fig 3C). At steady condition, NKT cells were the major cells expressing CXCR6 in the liver, whereas CD4^+^ T cells were the major cells expressing CXCR6 following infection (Fig 3D). Phenotypically, CXCR6^+^CD4^+^ T cells were exclusively CD44^+^CD62L^-^ cells (effector memory T cells) (Fig 3E) as well as IFN-γ-producing cells (Fig 3F). CXCR6^+^CD4^+^ T cells were the major sub-population of IFN-γ producing CD4^+^ T cells (Fig 3F). Collectively, those data demonstrated that CXCR6^+^CD4^+^ T cells were predominantly recruited to the liver following infection with *T*. *brucei*.

### Disruption of CXCR6 signaling prolongs survival of infected mice

**Fig 4:**
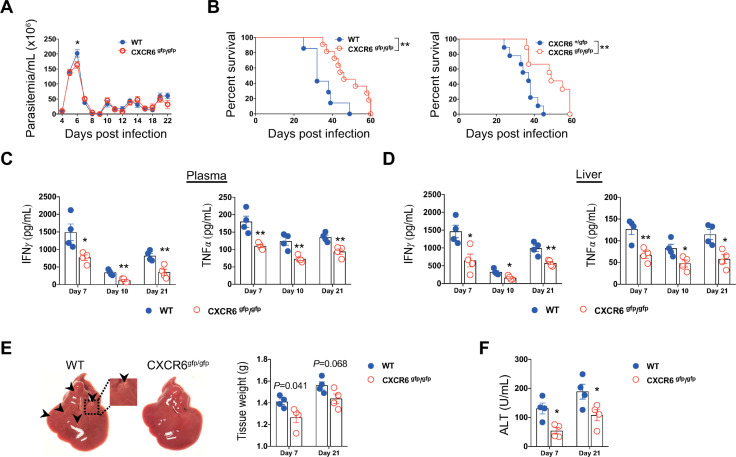
Genetic deletion of CXCR6 prolongs survival of mice infected with *T*. *brucei*. **A** The parasitemia of the WT and CXCR6^gfp/gfp^ mice infected with *T*. *brucei*. **B** The survival rate of WT, CXCR6^+/gfp^ mice, and CXCR6^gfp/gfp^ mice infected with *T*. *brucei*. **C** Plasma levels of IFN-γ and TNF-α in WT and CXCR6^gfp/gfp^ mice at indicated time-points post infection. **D** Levels of IFN-γ and TNF-α in supernatants of the cultured liver cells of WT and CXCR6^gfp/gfp^ mice at indicated time-points post infection. **E** (Left) Representative images of livers of WT or CXCR6^gfp/gfp^ mice on day 21 post infection, arrow heads indicate liver lesion. (Right) Quantification of the liver weight of WT or CXCR6^gfp/gfp^ mice at the indicated time-points post infection. **F** The plasma level of alanine aminotransferase (ALT) of WT or CXCR6^gfp/gfp^ mice at indicated time-points post infection. n = 7-11(**A**), 7–11 (Left, **B**), 9 (Right, **B**), and 4 (**C-F**), data are expressed as mean ± SEM from 2 independent experiments, compared by log-rank test (**B**), multiple *t* test (**A, C-F**). Dots represent biological replicates. *p< 0.05, and **p< 0. 01.

The substantial accumulation of CXCR6^+^CD4^+^ T cells in the liver during infection prompted us to examine the role of CXCR6 signaling during infection. Although CXCR6 deficient (CXCR6^gfp/gfp^) mice displayed slightly lower parasitemia at the peak of parasitemia, both WT and deficient mice could control the first wave of parasitemia. Consistent with previous observation [45], the parasitemia of both WT and deficient mice increased at the late stage of infection (S2 Fig). Overall, WT and CXCR6 deficient mice had a comparable parasitemia during infection (Figs 4A and S2). However, infected CXCR6 deficient mice survived significantly longer than infected WT and CXCR6^+/gfp^ mice, indicative of a detrimental role of CXCR6 signaling (Fig 4B). The prolonged survival of infected CXCR6 deficient mice correlated with significantly reduced plasma levels of IFN-γ and TNF-α and their secretion in the liver (Fig 4C and 4D). Compared to infected CXCR6 deficient mice, infected WT mice exhibited significantly higher liver weight with abundant pale dots highly suggestive of liver necrosis (Fig 4E). Moreover, deficiency of CXCR6 led to reduced plasma levels of ALT during infection (Fig 4F). Collectively, those data suggested that genetic ablation of CXCR6 enhanced the survival of infected mice, associated with attenuated inflammation and diminished liver injury.

### CXCR6 signaling mediates CD4^+^ T cell recruitment to the liver in a cell-intrinsic manner during infection

**Fig 5:**
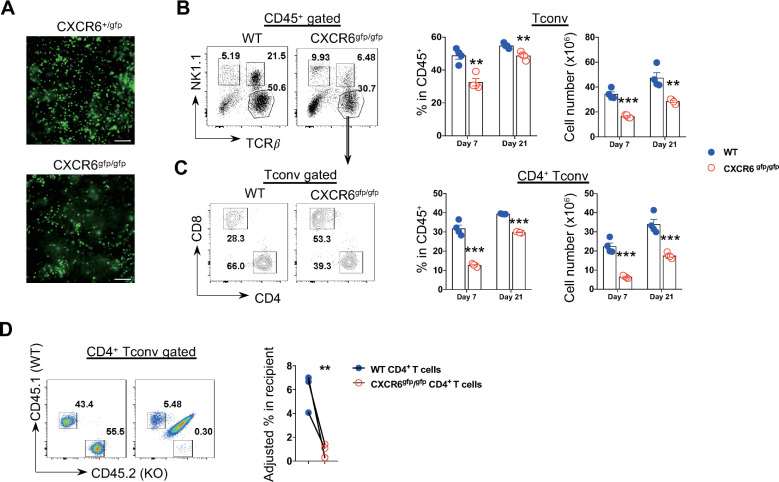
CXCR6 signaling mediates CD4^+^ T cell recruitment to the liver in a cell-intrinsic manner during infection. **A** Representative intravital images comparing the abundance of CXCR6-GFP cells in CXCR6^+/gfp^ and CXCR6^gfp/gfp^ mice on day 21 post infection. Scale bar, 200 μm. **B, C** The abundance of the intrahepatic conventional T cells (Tconv) (**B**) or CD4^+^ Tconv (**C**) in WT and CXCR6^gfp/gfp^ mice at indicated time-points of infection. (Left) Representative plots of day 7 post infection. (Right) Quantification of the frequency (within the CD45^+^ cells) or the absolute number. **D** The cell intrinsic requirement of CXCR6 in CD4^+^ T cell recruitment to the liver during infection. CD4^+^ T cells isolated from WT (CD45.1^+^) and CXCR6^gfp/gfp^ (CD45.2^+^) mice were mixed (at 1:1 ratio) and co-transferred intravenously to the recipient CD45.1^+^/2^+^ WT mice on day 0 post infection, and then the percentages of the transferred cells within the recipients were assessed on day 7 post infection. (Left) Representative plots showing the relative percentages of the co-transferred cells. (Right) Bar graph comparing the percentages of co-transferred CD4^+^ T cells within the recipients (adjusted to the initial ratio prior to the transfer). n = 3 (**A**), 4 (**B-D**), data are expressed as mean ± SEM from 2 independent experiments, compared by unpaired 2-tailed *t* test (**B, C**), or paired 2-tailed *t* test (**D**). Dots represent biological replicates. **p< 0.01, and ***p< 0.001.

We next examined how disruption of CXCR6 signaling affected liver immunopathology and the mortality of infected mice. Intravital imaging revealed that CXCR6^gfp/gfp^ (CXCR6 deficient) mice displayed fewer CXCR6-GFP cells in the liver compared to CXCR6^+/gfp^ mice during infection (Fig 5A), indicative of impaired recruitment of CXCR6^+^ cells in the absence of CXCR6 signaling. As CXCR6 can be expressed on various subsets of lymphocytes including NK cells, NKT cells, CD4^+^ and CD8^+^ T cells [31] (Fig 3A), we compared the recruitment of those cells in the liver between CXCR6 deficient and WT mice. The results showed that disruption of CXCR6 signaling did not affect the absolute number of NK cells during infection (S3 Fig). Despite reduced NKT cells in the liver of CXCR6 deficient mice at the early stage of infection, there was no significant difference in the absolute number of NKT cells between CXCR6 deficient and WT mice at the late stage of infection (S3 Fig). Importantly, CXCR6 deficient mice displayed significantly lower frequency and absolute number of T cells (Fig 5B), especially CD4^+^ T cells (Fig 5C) but not CD8^+^ T cells (S3 Fig) in the liver at both early and late stages of infection, demonstrating that CXCR6 signaling drove accumulation of CD4^+^ T cells in the liver. Disruption of CXCR6 signaling did not affect the survival, exhaustion, and proliferation of CD4^+^ T cells (S4A–S4C Fig). We next mixed CD45.1^+^ WT and CD45.2^+^ CXCR6 deficient CD4^+^ T cells (at 1:1 ratio) and co-transferred the cells to CD45.1^+^/2^+^ WT mice during infection. We found that there were significantly fewer transferred CXCR6 deficient CD4^+^ T cells in the liver of recipient mice compared to WT CD4^+^ T cells (Fig 5D), demonstrating that CXCR6 signaling mediated CD4^+^ T cell recruitment to the liver in a cell-intrinsic manner during infection.

### CXCR6^+^CD4^+^ T cells promote mortality of infected mice

**Fig 6:**
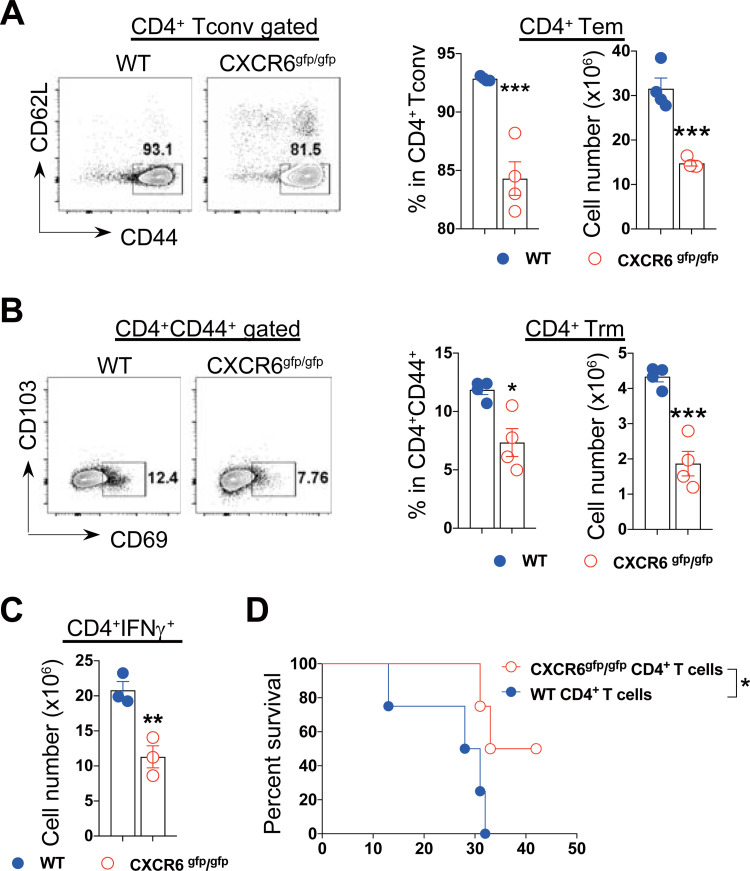
CXCR6^+^CD4^+^ T cells promote mortality of mice infected with *T*. *brucei*. **A** The abundance of the intrahepatic CD44^+^CD62L^-^CD4^+^ T cells (effector memory T cells, Tem) in WT and CXCR6^gfp/gfp^ mice on day 21 post infection. Cells were gated on CD4^+^ Tconv cells (CD45^+^TCRβ^+^NK1.1^-^CD4^+^). (Left) Representative plots. (Right) Quantification of the frequency and the absolute number. **B** The abundance of the intrahepatic CD44^+^CD69^+^CD4^+^ T cells (resident memory T cells, Trm) in WT and CXCR6^gfp/gfp^ mice on day 21 post infection. Cells were gated on CD4^+^CD44^+^ cells (CD45^+^TCRβ^+^NK1.1^-^CD4^+^CD44^+^). (Left) Representative plots. (Right) Quantification of the frequency and the absolute number. **C** The absolute number of IFN-γ producing CD4^+^ T cells in the livers of WT and CXCR6^gfp/gfp^ mice on day 21 post infection. **D** The survival rate of infected CXCR6^gfp/gfp^ mice receiving 5x10^6^ CD4^+^ T cells isolated from naïve WT or CXCR6^gfp/gfp^ mice on day 0 post infection. n = 4 (**A, B, D**), 3 (**C**), data are expressed as mean ± SEM from 2 independent experiments, compared by unpaired 2-tailed *t* test (**A-C**), or log-rank test (**D**). Dots represent biological replicates. *p< 0.05, **p< 0.01, and ***p< 0.001.

We further evaluated whether CXCR6 signaling had an impact on the phenotype and functionality of the recruited CD4^+^ T cells. The results showed that infected CXCR6 deficient mice exhibited significantly lower frequency and absolute number of CD44^+^CD62L^-^CD4^+^ T cells (Fig 6A) as well as CD69^+^CD103^-^ CD4^+^ T cells (Fig 6B) in the liver compared to infected WT mice, suggesting that CXCR6 signaling promoted CD4^+^ T cell transition to effector memory cells as well as resident memory cells. Moreover, infected CXCR6 deficient mice showed significantly lower absolute number of IFN-γ-producing CD4^+^ T cells in the liver (Fig 6C) despite unaltered frequency of those cells (S4D Fig). By contrast, deficiency of CXCR6 did not affect the transition of CD8^+^ T cells to resident memory cells (S4E Fig) or the absolute number of IFN-γ-producing CD8^+^ T cells (S4F Fig). Importantly, infected CXCR6 deficient mice receiving transferred WT CD4^+^ T cells survived significantly shorter than those mice receiving transferred CXCR6 deficient CD4^+^ T cells (Fig 6D), demonstrating that highly activated CXCR6^+^CD4^+^ T cells mediated early mortality of infected mice.

## Discussion

Tissue resident macrophages including KCs, microglia, and alveolar macrophages are a diverse population of leukocytes that originate from fetal monocytes during embryogenesis and reside in mammalian tissues, playing a prominent role in host defense and tissue homeostasis [7–9]. Previously, we have shown that liver macrophages play an essential role in intravascular clearance of African trypanosomes through phagocytosis of the parasites [4,46]. In the current study, we show that liver macrophages underwent cell death during *T*. *brucei* infection. Liver macrophages necroptosis has been previously observed during *L*. *monocytogenes* infection [12]. As a facultative intracellular pathogen, *L*. *monocytogenes* can survive and grow within macrophages and kill macrophages in a listeriolysin O- and type 1 IFN-dependent manner [12,47,48]. In contrast, *T*. *brucei* is unable to survive within liver macrophages [4,46]. The mechanism involved in liver macrophage depletion during *T*. *brucei* infection is unknown. It deserves further investigation.

Published work has shown that KCs are rapidly repopulated following depletion by diphtheria toxin and cell death caused by *L*. *monocytogenes* infection [10,12]. The repopulated KCs are genetically and functionally identical to the embryonic KCs [10]. Interestingly, mice infected with *L*. *monocytogenes* benefit from the repopulated KCs due to their strong antimicrobial effects and subsequent repair function [12]. In consistent with previous studies, repopulation of liver macrophages occurred in mice infected with *T*. *brucei*. Of note, three populations of macrophages were detected in the liver of infected mice. It is likely they belong to one population at different stages of maturation to give rise to fully differentiated KCs. Interestingly, the process of cell death and repopulation of live macrophages was accompanied by a substantial accumulation of CXCR6^+^CD4^+^ T cells in the liver during infection. Although we found repopulated macrophages secrete CXCL16, the detailed mechanism linking the replenishment of liver macrophages and accumulation of CXCR6^+^CD4^+^ T cells is unknown. We have previously shown that liver macrophages (upon internalization of trypanosomes) are highly activated [49]. It is likely that resident and/or repopulated macrophages attract CXCR6^+^CD4^+^ T cells through direct secretion of chemokines (e.g., CXCL16/CXCL9-11) and/or indirect activation of sinusoidal endothelial cells [50,51].

The finding that CXCR6 deficient mice infected with *T*. *brucei* had reduced liver injury and longer survival is of interest, which demonstrates that CXCR6 signaling is detrimental in our experimental setting. Association of CXCR6/CXCL16 axis with disease progression has been previously reported in autoimmune diseases and liver inflammation. For example, anti-CXCL16 antibody treatment attenuated rheumatoid arthritis in a mouse model [52]; accordingly, CXCR6 deficient mice exhibited a reduced incidence of arthritis and disease severity compared to WT mice, associated with diminished T cell cytokine polarization and T cell recruitment to inflamed joints [53]. In a mouse model of liver inflammation, deficiency of CXCR6 protected the mice from liver fibrosis progression; adoptive transfer of WT NKT cells, but not CD4^+^ T cells, restored hepatic fibrosis in CXCR6 deficient mice [39]. In addition, it has been recently reported that deficiency of CXCR6 signaling led to a better control of the bacterial burden in the lung during pulmonary infection with *M*. *tuberculosis* and that mice lacking CXCR6 displayed a reduced weight loss after acute influenza infection [54]. Interestingly, this improved disease progression was associated with reduced Th1-cytokine responses in the lung parenchyma [54]. However, CXCR6 deficiency did not affect the recruitment of CD4^+^ and CD8^+^ T cells to the lung during those infections; thus, the mechanism is not clear [54]. In contrast to the detrimental role of CXCR6 signaling, a beneficial role has been recently reported in liver cancer models [55,56]. In this model, CXCR6 is required to inhibits hepatocarcinogenesis by driving intrahepatic accumulation of NKT cells and/or CD4^+^ T cells for clearance of senescent hepatocytes [55] or inhibition of liver-selective tumors [56]. Thus, CXCR6/CXCL16 axis can play both detrimental and beneficial roles, depending on disease settings.

IFN-γ and proinflammatory cytokines such as IL-12 and TNF-α mediates protection in African trypanosomiasis [18,20,57,58]. Of note, IL-12 drives secretion of IFN-γ which promotes macrophage activation [57]. TNF-α is crucial for nitric oxide-mediated trypanosome killing [58]. However, inflammation must be tightly controlled and excessive secretion of IFN-γ causes fatal liver pathology and early mortality of mice infected with African trypanosomes [26,59]. In addition, early studies have shown that trypanosome-mediated immunopathological features were greatly reduced in infected TNF-α deficient mice [60]. It is noteworthy that infected CXCR6 deficient mice secrete significantly lower IFN-γ and TNF-α compared to infected wild-type mice. It is likely that the reduced production of IFN-γ and TNF-α contributes to the enhanced survival of infected CXCR6 deficient mice without compromising the parasitemia control. Although IFN-γ is produced by various subsets of lymphocytes [61], we have previously shown that excessive secretion of IFN-γ by CD4^+^ T cells mediates early mortality of susceptible mice [25]. In the current study, we show that infected CXCR6 deficient mice receiving WT CD4^+^ T cells survived significantly shorter than those receiving CXCR6 deficient CD4^+^ T cells, identifying CXCR6^+^CD4^+^ T cells as the pathologic CD4^+^ T cells that mediate early mortality of mice infected with African trypanosomes.

In summary, liver macrophages underwent cell death and were repopulated during *T*. *brucei* infection, associated with substantial accumulation of CXCR6^+^CD4^+^ T cells. CXCR6 signaling mediated CD4^+^ T cell recruitment to the liver in a cell-intrinsic manner during infection. CXCR6^+^CD4^+^ T cells mediate the early mortality of mice infected with *T*. *brucei*.

## Materials and methods

### Ethics statement

The study was performed in strict accordance with the recommendations in the Guide for the Care and Use of Laboratory Animals of the National Institutes of Health. The protocols of mouse studies were approved by the Institutional Animal Care and Use Committee (IACUC, file# R-SEPT-18-50) of University of Maryland, College Park.

### Mice

WT C57BL/6 (CD45.1^+^ or CD45.2^+^) mice and outbred Swiss white (CD1) mice were purchased from the National Cancer Institute (Frederick, MD). Breeding pairs of CXCR6^gfp/gfp^ mice (CXCR6^-/-^, stock# 005693) [33] in C57BL/6 background were purchased from the Jackson Laboratory. CXCR6^+/gfp^ mice were generated by crossing WT mice with CXCR6^gfp/gfp^ mice. CD45.1^+^/2^+^ WT mice were produced by crossing CD45.1^+^ WT mice with CD45.2^+^ WT mice. All mice were used at the age of 8- to 12-week.

### Parasites and infections

*T*. *brucei* AnTat1.1E was used in this study and the parasite was provided by Dr. Stefan Magez (Vrije Universiteit Brussels, Belgium). We used CD1 mice for passages of the parasites. CD1 mice were immunosuppressed with cyclophosphamide before intraperitoneal injection of the parasites. We took the blood of infected mice 3 days after infection and purified the parasites from the blood using DEAE-cellulose chromatography as described previously [4]. Mice were intraperitoneally infected with 5×10^3^ *T*. *brucei* AnTat1.1E.

### Antibodies and chemicals

Anti-mouse CD16/CD32 (Clone 2.4G2), CD45 (30-F11), CD45.1 (A20), CD45.2 (104), MHC II (M5/114.15.2), F4/80 (BM8), Clec4F (3E3F9), Tim4 (RMT4-54), CD11b (M1/70), Ly6C (HK1.4), TCR-β (H57-597), NK1.1 (PK136), CD4 (GK1.5), CD8 (53–6.7), CXCR6 (SA051D1), CD44 (IM7), CD62L (MEL-14), CD69 (H1.2F3), CD103 (2E7), IFN-γ (XMG1.2), as well as Annexin V and 7-AAD were purchased from Biolegend. Anti-mouse CRIg (NLA14), Nr4a1 (12.14) were purchased from ThermoFisher Scientific. CXCL16 (12–81) was purchased from BD Biosciences. ADAM10 (139712) was purchased from R&D Systems.

### Quantification of parasitemia and survival

To evaluate parasitemia, 1 μL of blood taking from the tail vein was diluted in 99 μL of sterile PBS and the parasites were counted at 40× magnification by phase-contrast microscopy. The survival time was defined as the number of days after infection that the infected mice remained alive.

### Purification of intrahepatic leukocytes

Intrahepatic leukocytes were purified as described previously [28]. Briefly, the liver was perfused with 10 mL sterile PBS and then processed on a gentleMACS Dissociator (Miltenyi). After collagenase IV digestion (100 U/mL) for 45 min at 37°C, the tissue homogenate was filtered through a 70-μm cell strainer in 50 mL RPMI-1640 medium containing 5% FCS. The cell suspension was centrifuged at 30g with the off-brake setting for 10 min at 4°C. The supernatant was collected and centrifuged at 300g for 10 min at 4°C. The pellet was resuspended in 10 mL 25% Percoll in HBSS and then centrifuged at 850g with the off-brake setting for 30 min at 23°C. A total of 0.5–1 mL ACK buffer was added to the cell pellets to lyse erythrocytes at room temperature for 5 min, followed by the addition of 14 mL RPMI-1640 medium containing 5% FCS. The cell suspension was centrifuged at 300g for 10 min at 4°C. Cells were resuspended in cold RPMI-1640 medium containing 5% FCS.

### Flow cytometry

Freshly isolated intrahepatic leukocytes were incubated with purified anti-mouse CD16/CD32 for 15 min in cold staining buffer to minimize unspecific antibody binding. For surface staining, cells were stained with specific fluorochrome-conjugated mAbs for 30 min at 4°C. After washes, cells were fixed and resuspended in staining buffer. For intracellular staining, fresh isolated cells were cultivated for 6 hours in presence of protein transport inhibitor (Brefeldin A) and incubated with mAb for 30 min at room temperature after fixation and permeabilization using the Intracellular Fixation and Permeabilization Buffer Set (Thermofisher Scientific). A final wash using the permeabilization buffer was performed before data acquisition. For apoptosis staining, following surface staining, cells without fixation were then stained with 7-AAD and Annexin V in Annexin V Binding Buffer (Biolegend) according to the manufacturer’s protocol. Data were collected using FACS Canto II or FACS Celesta (BD Biosciences) and analyzed with FlowJo (BD Biosciences).

### Determination of cytokines and alanine transaminase

The plasma levels of IFN-γ and TNF-α were determined with the ELISA kits from BD Biosciences or ThermoFisher Scientific. Liver alanine transaminase (ALT) activities were examined using EnzyChrom Alanine Transaminase Assay Kit (BioAssay Systems) according to the manufacturer’s instructions.

### Adoptive transfer of CD4^+^ T cells

CD4^+^ T cells were isolated from the spleen of naïve CD45.1^+^ WT and CXCR6^gfp/gfp^ (CD45.2^+^) mice using CD4 microbeads from Miltenyi Biotec. 5x10^6^ cells per phenotype were transferred via the tail vein on day 0 post infection. In one setting, CD45.1^+^ WT and CD45.2^+^ CXCR6 deficient CD4^+^ T cells were mixed (at 1:1 ratio) and co-transferred to CD45.1^+^/2^+^ WT mice to determine the cell intrinsic effects of CXCR6 on the intrahepatic recruitment of CD4^+^ T cells. In the other setting, WT and CXCR6 deficient CD4^+^ T cells were separately transferred to CXCR6^gfp/gfp^ mice to compare the recipients’ survival.

### Intravital microscopy

Intravital microscopy was performed as previously described [4,62]. In brief, mice were anaesthetized by intraperitoneal injection of a mixture of ketamine (200 mg/kg) and xylazine (10 mg/kg). Following anesthesia, cannulation of the tail vein was performed for the purpose of delivery of additional mixture of ketamine and xylazine during in vivo imaging. A lateral incision was made along the costal margin to the midaxillary line, and the liver was exposed. The mice were transferred to a customized acrylic imaging stage and the exposed liver was covered with a trimmed cover slip. During in vivo imaging, a heating lamp was used to maintain the body temperature of the mice and the liver was continuously moistened with a saline soaked Kimwipe. Videos or images were taken on the liver using the Zeiss Axio Examiner Z1 system.

### Statistical analysis

Data are presented as the mean ± SEM. Statistical significance was determined by Student’s *t* test, analysis of variance (ANOVA), multiple *t* test, or a log-rank test for curve comparison using the GraphPad Prism 8.0 software. A *p* value of p<0.05 is considered statistically significant.

## Supporting information

S1 Fig The expression of ADAM10 and CXCL16 by liver macrophages during *T*. *brucei* infection. The expression of ADAM10 (A) and CXCL16 (B) by intrahepatic F4/80^hi^CD11b^int^ macrophages in WT mice on day 0 (naïve) or 21 post infection was measured by flow cytometric analysis. Their expression was determined using median fluorescent intensity (MFI). n = 3–4, data are expressed as mean ± SEM, compared by unpaired two-tailed *t* test. Dots represent biological replicates. ****p< 0.0001.
(TIFF)

S2 Fig The parasitemia of the WT and CXCR6^gfp/gfp^ mice at the late stage of infection with *T*. *brucei*. The parasitemia of the WT and CXCR6^gfp/gfp^ mice infected with *T*. *brucei* was determined at the late stage of infection. n = 10, data from two independent experiments were pooled and are presented as mean ± SEM, compared by multiple *t* test.
(TIFF)

S3 Fig Recruitment of NKT, NK, and CD8^+^ T cells in WT and CXCR6^gfp/gfp^ mice during *T*. *brucei* infection. Quantification of the percentages and the absolute numbers of the indicated cells on day 7 and 21 post infection. CD8^+^ Tconv cells were gated as CD45^+^TCRβ^+^NK1.1^-^CD8^+^. n = 4, data are expressed as mean ± SEM, compared by unpaired 2-tailed *t* test. Dots represent biological replicates. ns: not significant, **p< 0.01, and ***p< 0.001.
(TIFF)

S4 Fig Characterization of CD4^+^ and CD8^+^ T cells in WT and CXCR6^gfp/gfp^ mice during *T*. *brucei* infection. **A** The frequency of apoptotic and necrotic death of intrahepatic CD4^+^ conventional T cells (Tconv, gated as CD45^+^TCRβ^+^NK1.1^-^CD4^+^) in WT mice and CXCR6^gfp/gfp^ mice on day 21 post infection. (Left) Representative plots. (Right) Quantification of the percentages. **B** The expression of PD-1 by intrahepatic CD4^+^ Tconv cells in WT and CXCR6^gfp/gfp^ mice on day 21 post infection. (Left) A representative plot. (Right) Quantification of the median fluorescent intensity (MFI). **C** The frequency of Ki-67^+^ cells within CD4^+^ Tconv cells in the livers of WT mice and CXCR6^gfp/gfp^ mice on day 21 post infection. (Left) Representative plots. (Right) Quantification of the frequency. **D** The frequency of IFN-γ^+^ cells within total intrahepatic CD4^+^ T cells in WT and CXCR6^gfp/gfp^ mice on day 21 post infection. (Left) Representative plots. (Right) Quantification of the frequency. **E** The abundance of the intrahepatic CD44^+^CD69^+^ (resident memory T cells, Trm) CD8^+^ T cells in WT and CXCR6^gfp/gfp^ mice on day 21 post infection. (Left) Representative plots showing the frequency. (Right) Quantification of the frequency within the indicated cell populations. Cells were gated on CD8^+^CD44^+^ cells (CD45^+^TCRβ^+^NK1.1^-^CD8^+^CD44^+^). **F** The abundance of the IFN-γ producing CD8^+^ T cells in the livers of WT and CXCR6^gfp/gfp^ mice on day 21 post infection. CD8^+^ Tconv cells were gated as CD45^+^TCRβ^+^NK1.1^-^CD8^+^. (Left) Representative plots showing the frequency. (Right) Quantification of the frequency and the absolute number. n = 4 (**A-C, E**), 3 (**D, F**), data are expressed as mean ± SEM, compared by unpaired two-tailed *t* test. Dots represent biological replicates. ns: not significant.
(TIFF)
