# Supplementary figures and images for: CXCR6+CD4+ T cells promote mortality during Trypanosoma brucei infection
Source: PLoS Pathog. 2021 Oct 6;17(10):e1009968. doi: 10.1371/journal.ppat.1009968 (PMC8523071; doi:10.1371/journal.ppat.1009968)

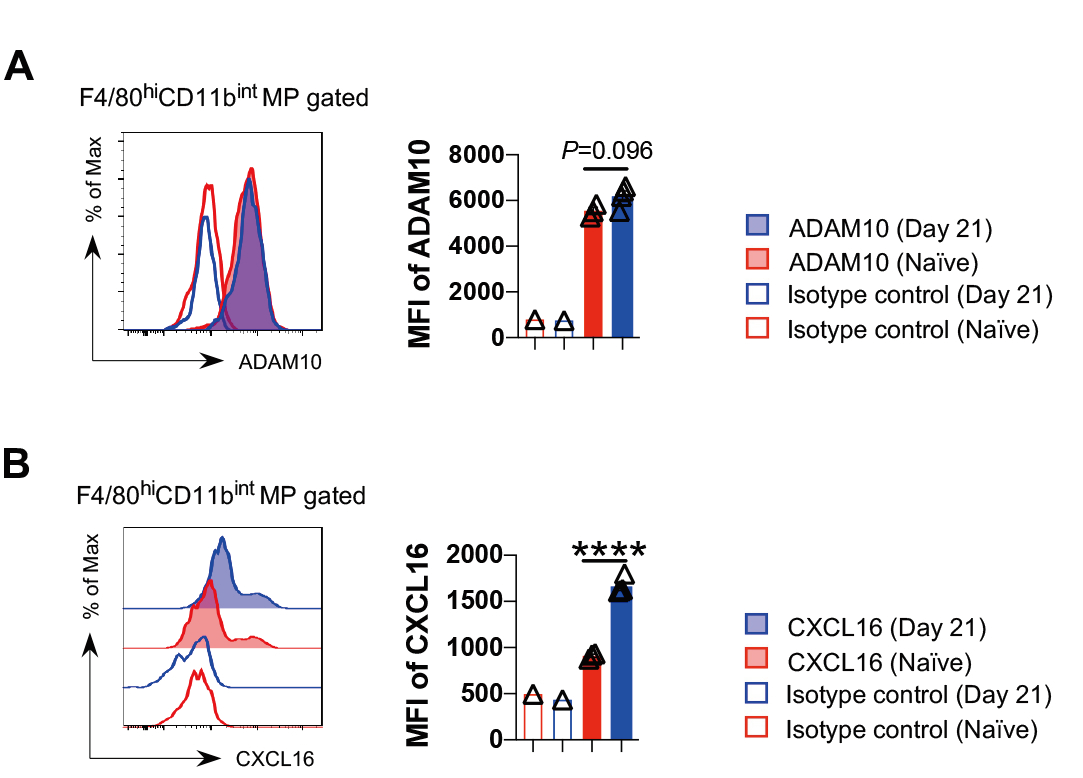

Supplement: S1 Fig — The expression of ADAM10 (A) and CXCL16 (B) by intrahepatic F4/80hiCD11bint macrophages in WT mice on day 0 (naïve) or 21 post infection was measured by flow cytometric analysis. Their expression was determined using median fluorescent intensity (MFI). n = 3–4, data are expressed as mean ± SEM, compared by unpaired two-tailed t test. Dots represent biological replicates. ****p< 0.0001. (TIFF) [file ppat.1009968.s001.tiff]

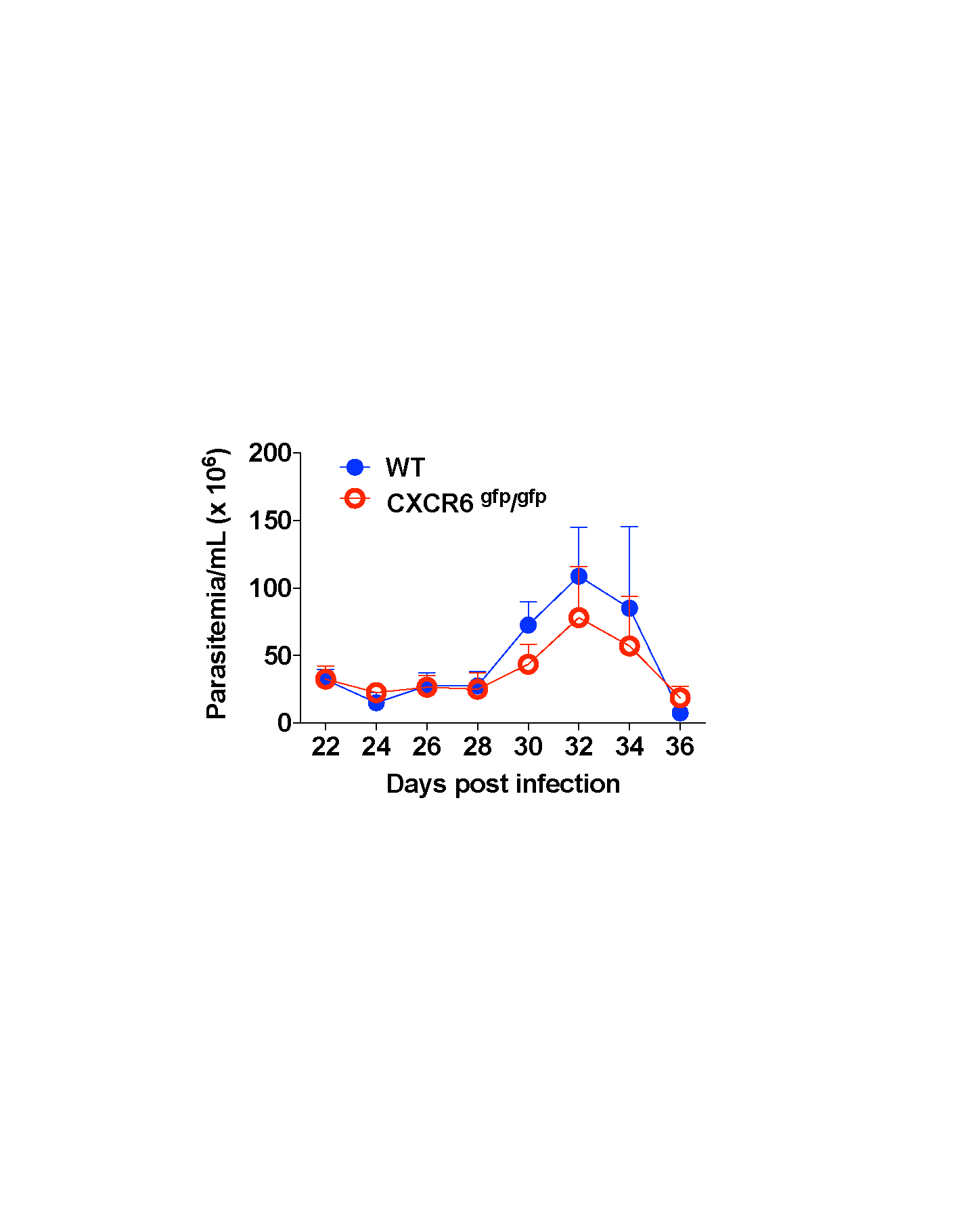

Supplement: S2 Fig — The parasitemia of the WT and CXCR6gfp/gfp mice infected with T. brucei was determined at the late stage of infection. n = 10, data from two independent experiments were pooled and are presented as mean ± SEM, compared by multiple t test. (TIFF) [file ppat.1009968.s002.tiff]

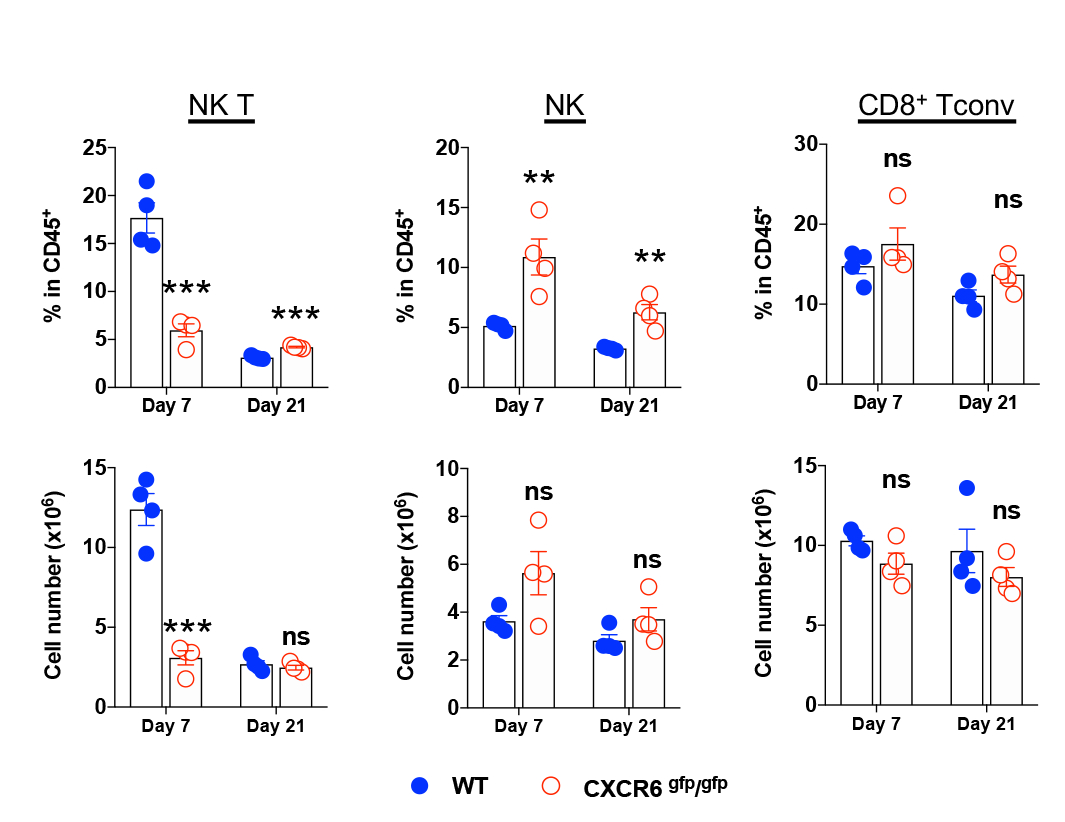

Supplement: S3 Fig — Quantification of the percentages and the absolute numbers of the indicated cells on day 7 and 21 post infection. CD8+ Tconv cells were gated as CD45+TCRβ+NK1.1-CD8+. n = 4, data are expressed as mean ± SEM, compared by unpaired 2-tailed t test. Dots represent biological replicates. ns: not significant, **p< 0.01, and ***p< 0.001. (TIFF) [file ppat.1009968.s003.tiff]

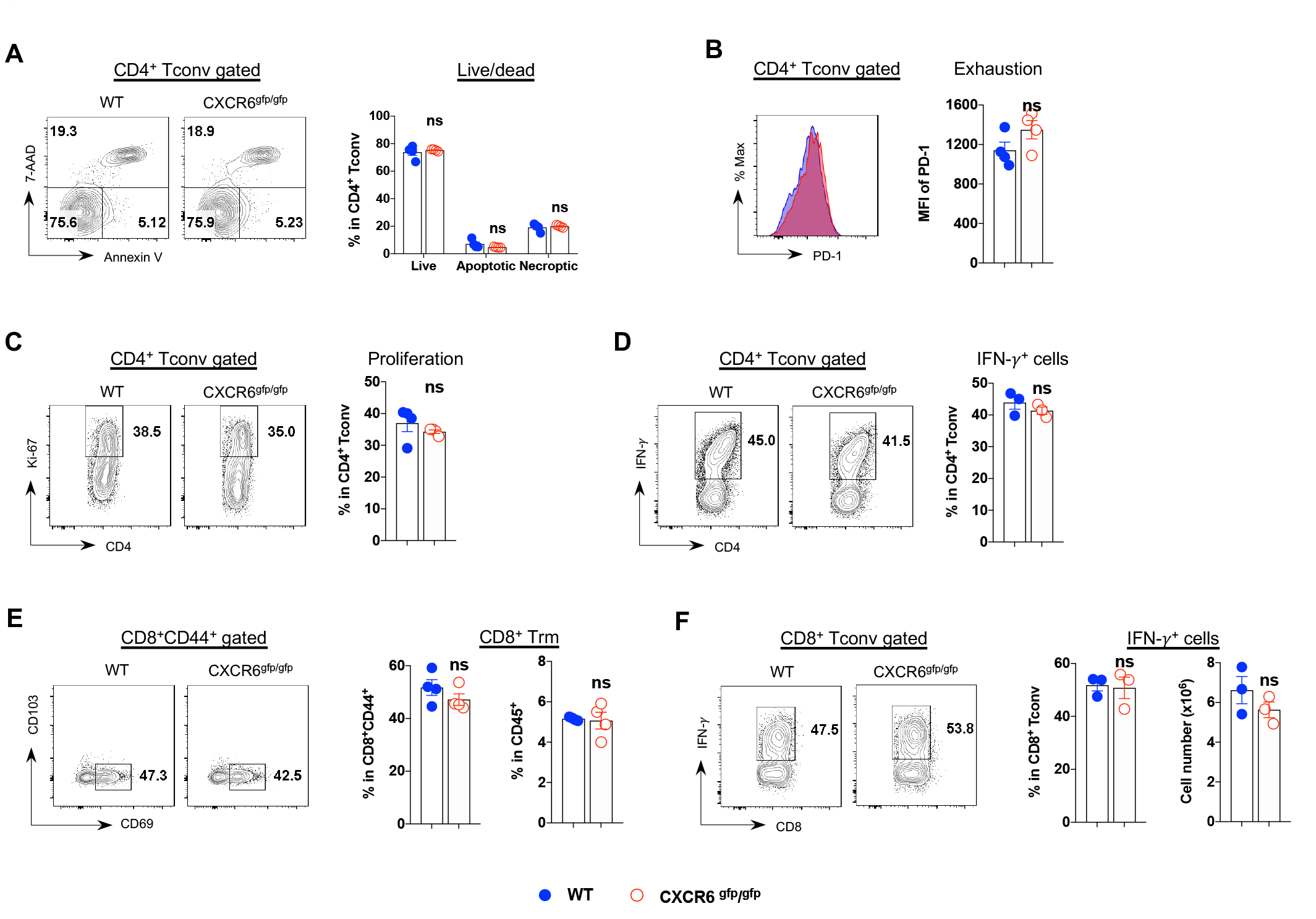

Supplement: S4 Fig — A The frequency of apoptotic and necrotic death of intrahepatic CD4+ conventional T cells (Tconv, gated as CD45+TCRβ+NK1.1-CD4+) in WT mice and CXCR6gfp/gfp mice on day 21 post infection. (Left) Representative plots. (Right) Quantification of the percentages. B The expression of PD-1 by intrahepatic CD4+ Tconv cells in WT and CXCR6gfp/gfp mice on day 21 post infection. (Left) A representative plot. (Right) Quantification of the median fluorescent intensity (MFI). C The frequency of Ki-67+ cells within CD4+ Tconv cells in the livers of WT mice and CXCR6gfp/gfp mice on day 21 post infection. (Left) Representative plots. (Right) Quantification of the frequency. D The frequency of IFN-γ+ cells within total intrahepatic CD4+ T cells in WT and CXCR6gfp/gfp mice on day 21 post infection. (Left) Representative plots. (Right) Quantification of the frequency. E The abundance of the intrahepatic CD44+CD69+ (resident memory T cells, Trm) CD8+ T cells in WT and CXCR6gfp/gfp mice on day 21 post infection. (Left) Representative plots showing the frequency. (Right) Quantification of the frequency within the indicated cell populations. Cells were gated on CD8+CD44+ cells (CD45+TCRβ+NK1.1-CD8+CD44+). F The abundance of the IFN-γ producing CD8+ T cells in the livers of WT and CXCR6gfp/gfp mice on day 21 post infection. CD8+ Tconv cells were gated as CD45+TCRβ+NK1.1-CD8+. (Left) Representative plots showing the frequency. (Right) Quantification of the frequency and the absolute number. n = 4 (A-C, E), 3 (D, F), data are expressed as mean ± SEM, compared by unpaired two-tailed t test. Dots represent biological replicates. ns: not significant. (TIFF) [file ppat.1009968.s004.tiff]
